# Intra-pulp temperature increase of equine cheek teeth during treatment with motorized grinding systems: influence of grinding head position and rotational speed

**DOI:** 10.1186/1746-6148-10-47

**Published:** 2014-02-21

**Authors:** Silvia Haeussler, Matthias Luepke, Hermann Seifert, Carsten Staszyk

**Affiliations:** 1 Institute of Veterinary-Anatomy, -Histology and -Embryology, Faculty of Veterinary Medicine, Justus-Liebig-University Giessen, Frankfurter Str. 98, D-35392 Giessen, Germany; 2 Institute for General Radiology and Medical Physics, University of Veterinary Medicine Hannover, Bischofsholer Damm 15, D-30173 Hannover, Germany; 3 formerly Institute of Anatomy, University of Veterinary Medicine Hannover, Bischofsholer Damm 15, D-30173 Hannover, Germany

**Keywords:** Equine, Pulp, Teeth, Dental treatment, Temperature

## Abstract

**Background:**

In equine practice, teeth corrections by means of motorized grinding systems are standard procedure. The heat resulting from that treatment may cause irreparable damage to the dental pulp. It has been shown that a 5.5°C temperature rise may cause severe destruction in pulp cells. Hence, the capability to continuously form secondary dentine is lost, and may lead, due to equine-typical occlusal tooth abrasion, to an opening of the pulp cavity.

To obtain reliable data on the intra-pulp increase in temperature during corrective treatments, equine cheek teeth (CT) were modified in a way (occlusal surface smoothed, apical parts detached, pulp horns standardized) that had been qualified in own former published studies. All parameters influencing the grinding process were standardized (force applied, initial temperatures, dimensions of pulp horns, positioning of grinding disk, rotational speed). During grinding experiments, imitating real dental treatments, the time span for an intra-pulp temperature increase of 5.5°C was determined.

**Results:**

The minimum time recorded for an intra-pulp temperature increase of 5.5°C was 38 s in mandibular CT (buccal grinding, 12,000 rpm) and 70 s in maxillary CT (flat occlusal grinding, 12,000 rpm). The data obtained showed that doubling the rotational speed of the disk results in halving the time span after which the critical intra-pulp temperature increase in maxillary CT is reached. For mandibular CT, the time span even drops by two thirds.

**Conclusion:**

The use of standardized hypsodont CT enabled comparative studies of intra-pulp heating during the grinding of occlusal tooth surfaces using different tools and techniques. The anatomical structure of the natural vital hypsodont tooth must be kept in mind, so that the findings of this study do not create a deceptive sense of security with regard to the time-dependent heating of the native pulp.

## Background

Routine dental care of equine teeth is often limited to eliminating lingual or buccal enamel points and to the flattening of irregularities on the occlusal surface. Also, a reduction of hooks and ramps may be necessary [1-3]. To this effect, the use of motorized grinding tools, meanwhile available in various types, has proven successful [4]. The use of these powered instruments with, e.g. rotating discs, produces heat at the surface while removing tooth material. Whether this surface-heat may cause intra-dental heating resulting in possible pulp damage has not been taken into account sufficiently yet. Research studies on brachyodont teeth showed that a 5.5 C temperature rise in the pulp may cause cell damage [5].

The thermal damage of pulpal cells is of great significance for equine hypsodont teeth. One layer of dentine producing cells, the odontoblasts, forms the inner side of the dentine. In this position, the odontoblasts continuously produce secondary dentine and thus strengthen the dentine coat. Stimuli on the peripheral surfaces of the tooth, e.g. heat from grinding processes, induce the production of secondary or reparative (tertiary) dentine by odontoblasts. While the continuous production of secondary dentine is essential for preventing pulp exposure in the process of physiological dental abrasion, the formation of tertiary dentine is considered as a reparative mechanism secondary to pathological conditions aimed at the protection of vital pulp areas [6]. Thermal injury or destruction of the odontoblasts through excessive overheating while grinding tall teeth, large hooks and ramps may lead to an insufficient occlusal dentine cover and thereby facilitates invading pathological processes [5].

For this reason, special attention is paid to data on the actual heat propagation in hypsodont equine CT during corrective grinding of occlusal surfaces.

In a former published study, we established a measurement system that generated reproducible data of intra-pulp temperature increases when applying a defined heat quantity to the occlusal surfaces of specially primed CT. This measurement system is based on the use of modified macerated equine CT, with geometrically defined pulp horns and occlusal surfaces ground flat. Temperatures were recorded continuously for individual pulp positions [7].

The aim of the presented study was to collect data about time-dependent heat-transfer into the pulp cavities during reduction of mandibular and maxillary equine CT. The grinding positions were related to routine equine dental treatment and measurements were taken for specified pulp positions.

## Methods

### Material

All used teeth were collected after horses were euthanized for other than dental reasons unattached to this study. The occlusal surfaces of macerated equine CT (Triadan 07 to 10; maxillary: n = 42, mandibular: n = 18) were smoothed with a diamond grinding disk, keeping the removal of material to a minimum. To access the pulp horns, the reserve crowns were separated twenty millimeters apical and parallel to the occlusal surfaces, using a diamond separating disk (dentalvet, Arendsee, Germany). These pulp horns were enlarged to a diameter of 1.5 mm and drilled to a uniform distance of 5 mm to the occlusal surface. For technical reasons, the diameter of the grinding disc had to cover the whole occlusal surface, the maxillary CT were dissected. Successively two groups were formed. The first group (group 1, n = 22) was halved at right angle to the occlusal surface and used for selective grinding above individual pulp horns. The maxillary CT of the second group (group 2, n = 21) were separated specifically through the infundibula into fragments a (with pulp horns 1 and 2) and b (with pulp horns 3 to 5). These CT were used for planar grinding of occlusal surfaces and tooth edges. In the final evaluation, the data from tooth fragments belonging to group 2 were combined and analyzed. The mandibular CT (group 3, n = 18) were not divided. The mass of individually prepared CT was weighed with a 0.01 g accuracy using a compact scale (EMB, Kern & Sohn GmbH, Balingen). After end of preparation not all pulp cavities were useable for the measurements because of cracks, broken drill bits or opened pulp cavities through dividing process. Next, with the aid of a syringe and a needle (18 G), thermal conductive paste (KERATHERM® KP97, thermal conductivity 5 W/m^.^K, Kerafol GmbH, Germany) was injected air-bubble-free into the enlarged pulp horns. For recording of the temperature inside the pulp cavities, type K thermocouples nickel-chromium-nickel (B + B Thermal-Technik GmbH, Germany, measuring range -50°C to +260°C) were inserted into the pulp openings up the top of the drillings which ended with 5 mm distance to the occlusal surface. The calibration of the thermocouples was performed as a two-point calibration in meltwater (0°C) and in boiling water (100 C).

Prior to the actual measurement of a temperature increase in the sub-occlusal pulp areas during grinding of the prepared CT, a reference temperature curve was created for each prepared tooth using a hotplate. Each single modified CT was placed with the occlusal surface on the external heat source (150°C) and intra-dental temperature increase was recorded (see Haeussler et al. [7]). After a cooling period at room temperature (21°C ± 1°C), the individually prepared CT were fastened in a machine vise (Holex, Hoffmann Group, Baunschweig, Germany) with insulating slices of wool felt (Felt manufacturer Gebr. Roeders AG, Soltau, Germany), in order to provide free access to the occlusal surface and to approximately 10 mm of the tooth crown from the occlusal and buccal/lingual or buccal/palatinal sides.

**Figure 1:**
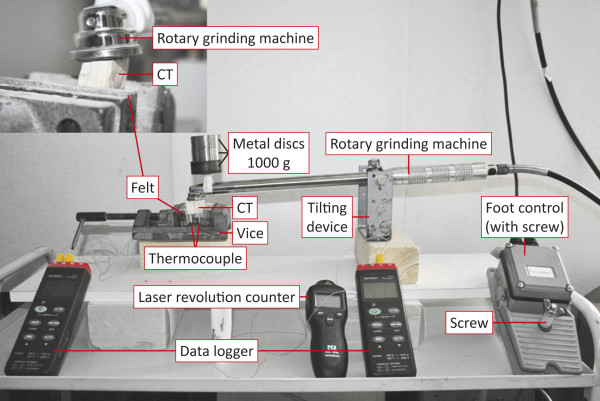
**Experiment set-up.** Measurement of the time required to reach the intra-pulp temperature increase of 5.5°C during the grinding experiments: Footswitch with setscrew to adjust the rotational speed of the drive motor, two thermal loggers to record temperatures, a laser-based optic revolution counter. The tooth model is fastened in a machine vise using insulating felt. Thermal sensors lead from intra-pulp to the thermal logger. The rotating diamond grinding disk can be adjusted to each individual tooth model so that maximum contact is guaranteed (see inset).

A motor-powered tooth grinding system with a rotating diamond grinding disk (PWS 500, dentalvet, Arendsee, Germany) was inserted into a specially for this study designed tilting device, which enabled the user to position the grinding disk in the required position on the occlusal surface or the tooth’s edge (see Figure 1). Metal disks were attached near the grinding head of the handpiece, producing a force of 1,000 g, thus simulating the average force applied during dental reduction with powered instruments. This force was determined during simulated tooth treatment by four external equine dentists, by means of an electronic precision scale (Kern EMB 600-2, Balingen, Germany). In the course of this simulation, these experts pressed the grinding system with its grinding head onto the scale exerting an estimated force. A setscrew at the electronic footswitch was used to limit the rotational speed of the driving motor (dentalvet, Arendsee, Germany) to 6,000 ± 50 rpm or 12,000 ± 50 rpm. The rotational speed was verified during each trial with the aid of a laser revolution counter (PCE-DT62, PCE Group GmbH, Arnsberg, Germany). At the starting point of the trials, the CT had a basic temperature of 20°C ± 0.5°C in the pulp openings. The trials were ended when a temperature of 30°C was reached at the sub-occlusal measurement points. Then, each tooth was cleaned with compressed air to remove dental debris from the grinding process. With the aid of a needle (18 G), the remaining heat conducting paste was extracted from the pulp horns and the removed mass was weighed using an electronic precision scale.

Temperatures were recorded with a data logger at 1 second intervals (VOLTCRAFT S309, Conrad Electronic SE, Germany). Subsequently the recorded data was analyzed determining the time for an intra-pulp temperature increase of 5.5°C. Therefore the duration of temperature increase from 24.5°C to 30.0°C was determined.

To verify the measurement system, an additional trial was carried out in each case (flat versus edge; 6,000 versus 12,000 rpm) using two compact marble blocks (19.7 × 17.3 × 23.1 mm^3^), whose measurements corresponded to those of an average CT specimen and which were equipped with two identical, diagonally drilled holes for the insertion of thermal sensors. Hole 1 was positioned at distances of 5.8, 5.8, 9.6 and 11.9 mm from the edges. Hole 2 was made at distances of 4.6, 4.8, 10.8 and 13.0 mm from the edges. The distance between the two holes measured 6.7 mm.

### The tooth-grinding experiment was carried out in two phases

#### Phase 1: selective grinding of occlusal surfaces above individual pulp positions

After establishing a standard curve with the aid of the above-mentioned heat application, the maxillary CT (group 1; n = 22) were ground at a rotational speed of 6,000 rpm. For this purpose, rotating grinding disks were positioned centrally above the individual pulp positions [8] and the time was measured until the temperature in the assigned sub-occlusal pulp area increased to 30°C. Next, the direct temperature rise after stopping the grinding process as well as the time span necessary to reach the basic temperature in the ground pulp positions were recorded.

#### Phase 2: planar grinding of occlusal surfaces and tooth edges

Since direct grinding above individual pulp positions is seldom practiced in equine dentistry, the grinding positions were modified. The individual pulp positions were defined [8] and analyzed. To evaluate the effects of the modified rotational speed of the motorized grinding system, the maxillary CT (group 2; n = 21) and the mandibular CT (group 3; n = 18) were divided into two sub-groups of identical size. Eleven CT of group 2 were ground at about 6,000 rpm and 10 CT at about 12,000 rpm. Of group 3, nine CT were ground at 6,000 rpm and another nine CT at 12,000 rpm. The following experiments were carried out successively with each specimen in groups 2 and 3:

1. The occlusal surfaces were heated to 150°C in order to establish a reference temperature curve.

2. The rotating grinding disk was positioned horizontally on the occlusal surface. This procedure simulated the flattening of cusps on the occlusal surface.

3. The rotating grinding disk was positioned either on the lingual CT edges of the mandibular teeth or on the buccal CT edges of the maxillary CT, at an angle of approximately 30° to the occlusal surface. This procedure simulated the grinding of sharp enamel points often found in these locations.

4. The rotating disk was either positioned on the buccal edge of the mandibular CT or on the palatine edge of the maxillary CT at an angle of approximately 30°. These grinding positions are rarely applied in practice.

5. The masses of the prepared CT were weighed before and after all grindings using an electronic precision scale in order to determine the difference in mass removal relative to the rotational speed used.

#### Statistics

The statistical evaluation of the data from the experiments was carried out with WinSTAT® for Microsoft® Excel. After a descriptive reprocessing of the data obtained, the unpaired *t*-test was conducted to ascertain significant deviations for the various pulp positions of the four CT groups. The five pulp positions were utilized as a dependent variable, whereas the rotational speeds of 6,000 and 12,000 rpm were considered independent grouping variables. The CT groups were divided into maxillary and mandibular CT as well as grouped by planar and edge grinding positions. P-values lower than 0.05 were considered statistically significant. In addition, the Wilcoxon test, a parameter-free test method for small samples, was utilized to test the two measurement series on an equal basis. The Bartlett test, which includes the Chi-square-test, was utilized to test for variance equality before carrying out the one-factor and two-factor analysis of variance with the grouping factors “position in upper and lower jaw” or ”varying rotational speeds of 6,000 and 12,000 rpm”.

## Results

**Figure 2:**
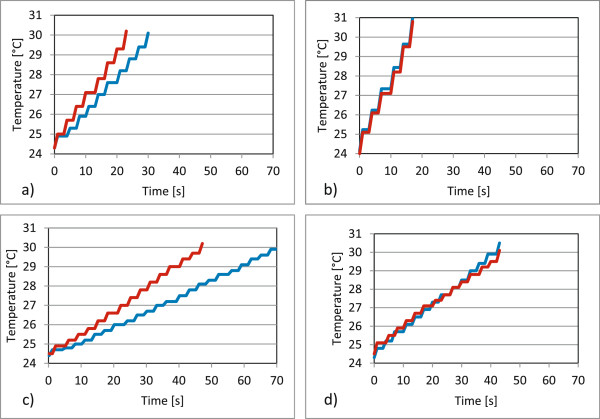
**Grinding of compact marble blocks.** The blue line marks the temperature increase in the drilling with the thermocouple that is farther away from the grinding head when grinding the edge of the marble block in graphic **a)** and **c)**. The red line represents the drilling with the thermocouple that is nearer to the grinding head when grinding the edge in graphic **a)** and **c)**. **a)** Grinding of edge at 12,000 rpm **b)** Grinding of flat surface at 12,000 rpm. **c)** Grinding of edge at 6,000 rpm **d)** Grinding of flat surface at 6,000 rpm.

Grinding the surface of two compact marble blocks resulted in a constant temperature increase at both measurement points. The time to reach the 30°C mark was shorter by 60% at 12,000 rpm compared to 6,000 rpm. Grinding the edge at 6,000 rpm resulted in a significant faster temperature increase (34%) in the hole drilled near the grinding head (47 s close to the grinding head compared to 71 s farther from the grinding head) and by 29% when working at 12,000 rpm (22 s close to the grinding head compared to 31 s farther from the grinding head) as well as by approximately 55% in both holes at increased rotational speed (see Figure 2).

### Phase 1: selective grinding of occlusal surfaces above the individual pulp positions

**Figure 3:**
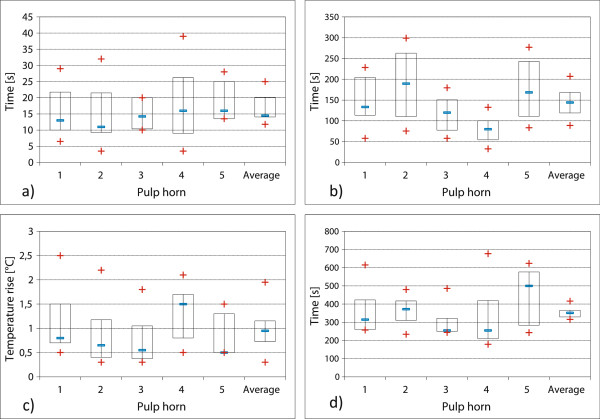
**Temperature rise in pulp horns of maxillary CT (group 1).** Temperature rise after application of hot plate and grinding the occlusal surface, respectively. Arithmetic mean values as well as their standard deviations are displayed. The short line within the rectangle shows the median. The upper and lower limits of the rectangle correspond to the 25th and 75th percentiles, respectively. Minimum and maximum values are marked with a + -symbol. The mean values of the individual pulp positions are displayed: pulp position 1: n = 13, pulp position 2: n = 13, pulp position 3: n = 6, pulp position 4: n = 9, pulp position 5: n = 5. **a)** Heat application by means of hotplate: measurement of intra-pulp temperature increase by 5.5°C. **b)** Selective grinding of supra-pulp occlusal surfaces. **c)** Intra-pulp temperature increase after ending the grinding process. **d)** Intra-pulp cooling phase: measurement of timespan until reaching the base temperature of 22°C.

The evaluation of maxillary CT (group 1) was performed separately for all measured pulp horns (n = 46). The time measured for the sub-occlusal temperature rise showed significant differences when working with an external heat source (hotplate, Figure 3a) or the actual tooth grinding method (Figure 3b). Using an external heat source (150°C), the time to increase the sub-occlusal pulp temperature by 5,5°C varied between 3.5 and 39 seconds in the individual pulp positions, i.e. a mean value of 16 seconds (median 13.5 s). During the grinding of specific occlusal surface sections, heating the sub-occlusal pulp area by 5.5°C took between 32.5 seconds (minimum) and 299 seconds (maximum), i.e. a mean value of 143 seconds (median 129.5 s). The average cooling period was 356 seconds (median 324 s, minimum 179 s, maximum 677 s, see Figure 3d) and the average temperature increase after stopping the grinding process was 1.0°C (median 0.7°C, minimum 0,3°C; maximum 2.5°C, see Figure 3c).

### Phase 2: planar grinding of occlusal surfaces and CT edges

1. During the application of heat (150°C), the maxillary CT (group 2) needed an average of 20.7 seconds (median 19.0 s) to reach an intra-pulp temperature increase of 5.5°C. The sub-occlusal area of pulp horn 5 warmed fastest; it took 17.9 seconds, while the heating of pulp horn 4 was slowest (23.5 seconds).

**Figure 4:**
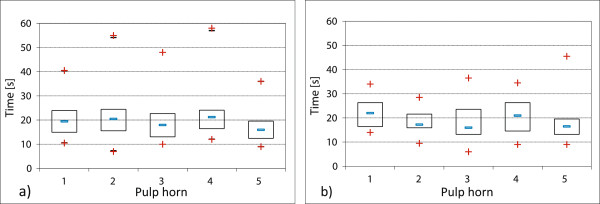
**Experiments with heat application (150°C).** Measurement of intra-pulp temperature increase by 5.5°C to test the experiment-setup (the whiskers mark the 5th and 95th percentiles). **a)** Maxillary CT (group 2: n = 21) **b)** Mandibular CT (group 3: n = 18).

For the mandibular CT (group 3), it took 19.4 seconds (median 18,5 s) on average to reach the critical temperature increase of 5.5°C after continuous contact with the heat source (minimum: 16.5 s; maximum: 21.2 s). No significant differences were noticed (P ≥ 0.05) in the heating of pulp horns 1 to 5 between CT groups 2 and 3 (see Figure 4).

2. On average, maxillary CT (group 2) grounded flat at 6,000 rpm, took 148 seconds (median: 130 s) for the intra-pulp heating of 5.5°C.

Maxillary CT (group 2), grounded flat at 12,000 rpm, took less time, i.e. 70 seconds on average (median: 68 s).

Mandibular CT (group 3), grounded flat at 6,000 rpm, reached the defined temperature increase after an average of 131 seconds (median 126 s).

On average, those mandibular CT (group 3) which were grounded at 12,000 rpm took 42 seconds for a temperature increase by 5.5°C (median: 41 s).

**Figure 5:**
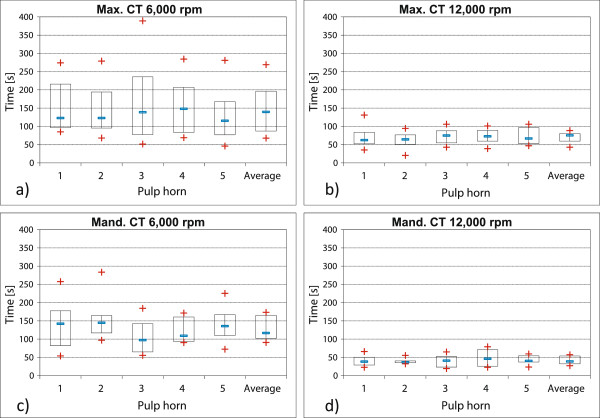
**Comparison of planar grinding of mandibular and maxillary CT at varying rotational speeds.** Intra-dental temperature rise after planar grinding of the occlusal surface. The mean values of the individual pulp positions are displayed in maxillary CT: pulp position 1: n = 20, pulp position 2: n = 20, pulp position 3: n = 16, pulp position 4: n = 20, pulp position 5: n = 19. In mandibular CT: pulp position 1: n = 18, pulp position 2: n = 18, pulp position 3: n = 17, pulp position 4: n = 16, pulp position 5: n = 17. **a)** Grinding of maxillary CT (group 2) at 6,000 rpm. **b)** Grinding of maxillary CT (group 2) at 12,000 rpm. **c)** Grinding of mandibular CT (group 3) at 6,000 rpm. **d)** Grinding of mandibular CT (group 3) at 12,000 rpm

The doubling of revolutions per minute thus led to a significant reduction of the time needed to reach the critical temperature in the core of the tooth (see Figure 5). The differences within the group of maxillary CT were significant with P-values lower than 0.05, while, the differences within the group of the mandibular CT groups were highly significant (P-values lower than 0.0003).

Neither at 6,000 rpm nor at 12,000 rpm, did individual pulp horns in maxillary or mandibular CT warm significantly faster. The P-values for both groups were consistently higher than 0.3.

3. When grinding the buccal edges of maxillary CT at 6,000 rpm, the heating of pulp horns 1 and 2 took comparatively longer than during the planar grinding (at average 323 s). Doubling the revolutions per minute to 12,000 rpm almost halved the time (at average 166 s) before the temperature increase of 5.5°C was reached.

Neither pulp horn showed significant faster heating (P ≥ 0.44).

During the grinding (6,000 rpm) of lingual edges of mandibular CT (group 3), pulp horn 3 was the first in which a temperature increase of 5.5°C was recorded after 139 s (average value). The time decreased by more than two thirds (to average 40 s) when doubling the rotational speed (12,000 rpm). Neither during the grinding of lingual mandibular tooth edges at 6,000 rpm, nor during the grinding at 12,000 rpm was a significantly faster heating of any specific pulp horn apparent (P ≥ 0.13).

**Figure 6:**
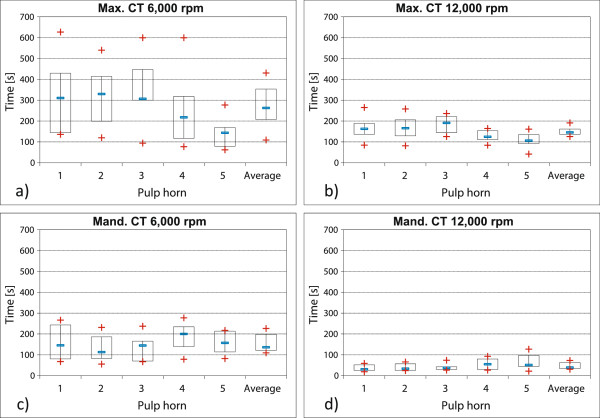
**Comparison of edge grinding of maxillary CT (group 2) and mandibular CT (group 3) at varying rotational speeds.** Data for maxillary CT pulp horns 1 (n = 20) and 2 (n = 20) were recorded during grinding the buccal tooth edge, data for pulp horns 3 (n = 16), 4 (n = 20) and 5 (n = 19) were collected during grinding the palatinal tooth edge. Data for mandibular CT pulp horns 1 (n = 18) and 2 (n = 18) were collected during grinding the buccal tooth edge, data for pulp horns 3 (n = 17), 4 (n = 16) and 5 (n = 17) were recorded during grinding the lingual tooth edge. **a)** Grinding of buccal and palatinal tooth edges of maxillary CT (group 2) at 6,000 rpm. **b)** Grinding of buccal and palatinal tooth edges of maxillary CT (group 2) at 12,000 rpm. **c)** Grinding of buccal and lingual tooth edges of mandibular CT (group 3) at 6,000 rpm. **d)** Grinding of buccal and lingual tooth edges of mandibular CT (group 3) at 12,000 rpm.

Also in this case, doubling the rotational speed led to a significantly shorter heating time, in maxillary CT (P ≤ 0.007) as well as in mandibular CT (P ≤ 0.002) (see Figure 6).

4. During the grinding of palatinal edges of maxillary CT (group 2; pulp horn 3 to 5) at 6,000 rpm, critical temperature increases were first measured in pulp position 5 (at average 140 s). Significant differences in the heating of pulp positions were recorded (P = 0,006).

After increasing the rotational speed to 12,000 rpm, these heating times became considerably shorter (at average 109 s for pulp horn 5). The heating of individual pulp horns varied significantly again (P = 0.0009).

During the grinding of buccal tooth edges of the mandibular CT (group 3) at rotational speeds of 6,000 and 12,000 rpm, no significantly faster heating of a single pulp horn (P ≥ 0.48) was recorded. But the average time of heating pulp horn 1 and 2 decreased from 144 s to 38 s (see Figure 6).

5. The average mass reduction in maxillary CT (group 2), grounded at 6,000 rpm, amounted to 0.62 g and to 1.11 g after grinding at 12,000 rpm. No significant differences were recorded in the mass loss at varying rotational speeds (P = 0.09).

**Figure 7:**
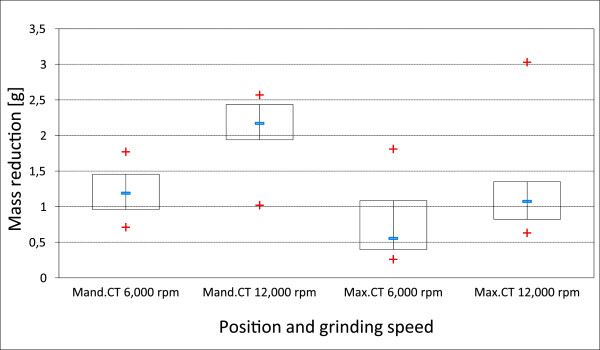
**Removed dental material.** Mass reduction after ending the grinding processes (planar, palatinal/lingual edge and buccal edge).

The mass of mandibular CT (group 3) diminished on average by 1.21 g (mean value), when using a rotational speed of 6,000 rpm and by 2.03 g (mean value) at a rotational speed of 12,000 rpm. The loss of mass was significantly higher in mandibular CT after doubling the rotational speed (see Figure 7).

## Discussion

### Phase 1: selective grinding of occlusal surfaces above individual pulp positions

In this study, the pulp cavities were standardized by drilling precise holes. Additionally a constant distance of 5 mm to the occlusal surface was defined. This artificially created distance of 5 mm is far smaller than the average distances determined in studies of native equine CT. Becker [9] states an average distance of 10 mm, whereas White and Dixon [10] measured average values of 7.5 mm to 12.8 mm on CT sawed open. Therefore, it was expected that the modified teeth utilized in this study would show very homogenous data because of the standardized distance of the top of the pulp to the occlusal surface. Despite the use of standardized teeth, the results depicted a broad time span when measuring the temperature increase within the pulp cavities during the grinding process above individual pulp positions. These results once again illustrate the large anatomical differences in equine CT. Although a constant distance between pulp and occlusal surface of 5 mm per drilled hole was defined for this study, it is possible that beyond the drilling area, further pulp expansions might have been present in some CT. In the macerated teeth, these pulp expansions would have been filled with heat conductive paste, resulting in increases of temperature occlusally-directed pulp expansions. Such pulp expansions getting close to the occlusal surface by less than 1 mm have been described for mandibular as well as maxillary CT [11]. Although occlusal sections of the pulp branches often narrow to less than 50 μm, they may nevertheless influence the heat conduction from the occlusal surface to the center of the tooth. While the actual distance between vital pulp and occlusal surface is a decisive factor for the heat conduction into the tooth, this distance is unfortunately very variable and unpredictable [10]. Also, the situation is different in teeth that deviate from the normal anatomic form and develop excessively overgrown teeth. Examinations of protuberant CT showed that in 49% (46/94) of these cases the dentine coverage above the individual pulp horns was less distinct than in regularly shaped teeth [12]. Consequently, the risk for iatrogenic heat damage during the grinding of dental overgrowth might be higher in comparison to routine treatments of only slightly altered teeth.

In a study by Van den Enden and Dixon [13], in 10% of 110 CT extracted due to apical infections, so-called occlusal pittings were discovered. The latter are microscopic cracks in the supra-pulpal column of secondary dentine. It seems likely that such columns of secondary dentine affected by these changes might display faster heat conduction into the pulp tissue underneath. Macroscopically, the teeth in this study did not show occlusal pittings, but because of their small size, their presence cannot be totally excluded. The necessary planar grinding of the occlusal surface in preparation for further treatment could also have led to micro-cracks in the occlusal dentine cover with the effect that heat propagation was sped up, if these cracks were filled with heat conductive paste. A prevalence of occlusal pittings in clinically inconspicuous equine teeth has not been reported. These findings cannot be easily recognized macroscopically. Endoscopic examinations (with a rigid endoscope) of the occlusal surfaces might aid in detecting occlusal pitting and dental fractures Prior to dental treatment, an rigid endoscope seems to be an appropriate tool for a thorough examination, especially of the caudal CT, that could detect a possible incomplete pulp coverage [2].

It is of great importance that the core temperature rose by 1°C on average, even after ending the grinding process. At first sight, this increase seems low, but in the presence of a potential damage of odontoblasts after a temperature increase of only 5.5°C, the heating of an additional 1°C after ending the grinding process is certainly noteworthy. The rate of the cooling process is also of interest. On average, cooling to the initial temperature takes more than twice as long as the temperature increase. The danger is that repeated grinding processes could take place in a phase when the pulp temperature has already risen above the normal value. However, it is assumed that intra-pulp blood circulation, the contact to neighboring teeth, the surrounding gingiva and bones with blood vessels and the moist film of saliva are factors supporting heat transmission after grinding treatments. Therefore, post treatment cooling might occur faster in vivo compared to experimental conditions. Nevertheless, it is important to develop grinding tools and techniques that provide optimized substance reduction at a minimized temperature increase. Based upon the present experimental setting (grinding with diamond grinding disks, force applied to the tooth = 1,000 g) the following recommendations for the grinding procedure can be given:

• The occlusal tooth surface should be checked thoroughly for physiological occlusal pulp closure

• The rotational speed should be low as possible.

• A grinding interval at one particular spot (e.g. one tooth) of maximum 30 seconds should not cause any pulp damage.

### Phase 2: planar grinding of occlusal surfaces and tooth edges

The grinding tests in phase 2 refered to therapeutic treatments of frequent findings in equine CT, such as sharp lingual and buccal enamel edges, as well as focal dental overgrowths of occlusal surfaces [1,2,14] with the limitation of a statically grinding process at one point.

Various authors performed temperature measurements in equine CT during the grinding process. All of them used teeth with untreated pulp cavities. The temperature sensors were positioned in the pulp horns [15,16], as well as in drill-holes positioned in the lateral hard tooth substances with defined distances to the occlusal surfaces [17].

Either extracted macerated mandibular CT [17], or extracted maxillary CT with original pulp were used [15,16].

During the measurement processes, dental material was removed from all teeth. Consequently, teeth used in the first studies were no longer available for follow-up and comparative studies. In this context, one should bear in mind that intra-pulp temperature measurements produce very heterogeneous results in non-primed equine teeth [7], due to the extremely variable anatomy of equine CT as to the distance between the pulp branches and occlusal surfaces. Therefore, comparative studies of pulp heating using various grinding tools and techniques prove only successful when standardized teeth are used to generate reproducible and consistent temperature measurements [7].

In contrast to the experiments by Allen et al. [17] and Wilson and Walsh [15] dealing with temperature measurements in equine teeth while grinding with rotating instruments with hard metal accessories, the present studies were carried out with diamond-coated grinding disks, which are offered now by a great number of manufacturers. While in other studies either mandibular CT [17] or maxillary CT [15,16] were used, it was focused on performing the measurements of this study in both upper and lower CT which display significant anatomical differences [6]. The force applied has a big influence on heat generation during the removal of tooth substances [18,19]. In this study, it was based on preliminary studies with various dental experts and remained constant at 1,000 g. In human dentistry, it has long been known that the rotational speed used during the removal of tooth material is another important parameter for the generation of heat in the core of the tooth [18]. Rotational speeds of 6,000 rpm and 12,000 rpm were chosen for the present study. Results of pilot studies on a variety of different rotational speeds indicated using 6.000 and 12.000 rpm respectively in this study.

In this study, the time span of an intra-pulp temperature increase of 5.5°C was measured, in order to gather data that could contribute to avoiding possible iatrogenic damage of the pulp caused by an excess of heat. This is a completely new approach. In all previously quoted studies on the heating of equine teeth, the grinding time was defined prior to the actual grinding process (15 and 20 seconds by Wilson and Walsh, [15]; 1 and 2 minutes by Allen et al., [17] and 30, 45, 60 and 90 seconds by O’Leary et al., [16]). The heating was measured afterwards. In order to avoid iatrogenic damage by the grinding processes, it makes more sense to determine the time it actually took to reach the critical increase of 5.5°C.

The application of approximately 150°C (±3°C) during pilot studies resulted in a uniform increase of temperature in all pulpa positions, in maxillary teeth as well as in mandibular teeth. The relatively high temperature of 150°C, which was applied to the entire surface may explain this. Since the influx of heat is, among other factors, proportional to the temperature decrease and the surface area, heating by means of a hotplate leads to a high heat influx into the tooth. The varying heat capacities inherent in differing masses of different tooth substances, as well as their differing surface sizes, do not play an important role; maxillary and mandibular CT heat up equally fast.

Grinding of the planar occlusal surface at approximately 6,000 rpm produced a relatively small heat influx into the tooth occurs. Now the critical temperature increase of 5.5°C was reached much faster (by 12%) in mandibular CT than in maxillary CT treated under the same conditions.

Doubling the rotational speed to 12,000 rpm shortened the time to reach the critical temperature increase in maxillary CT by 52% and even by 78% in mandibular CT. This result indicated that, on the one hand, the heat development in tooth surfaces was influenced to a great extent by the speed of the rotating grinding disk and, on the other hand, it points to the big differences in the heating of maxillary and mandibular CT. Reasons for these differences may be the differences in masses of maxillary and mandibular CT. This should not be neglected during equine dental treatment.

When simulating the grinding of buccal edges of maxillary teeth, doubling the rotational speed to 12,000 rpm resulted in a time reduction of the grinding interval by 50%.

Doubling the rotational speed for the grinding of lingual edges of mandibular CT led to a remarkable decrease of 70% of time span before the intra-pulp temperature increase reached 5.5°C.

In general, the grinding of palatinal tooth edges of maxillary teeth and buccal edges of mandibular CT is not considered a routine procedure in veterinary practice. However, in exceptional cases, e.g. when teeth are malpositioned, it may be necessary to undertake corrections. The difference in heating of the pulp positions in the maxillary CT is noteworthy and may be explained by the graded alignment of pulp positions from palatinal (pulp 5) to buccal (pulp 1 and 2) [6].

The grinding of buccal mandibular tooth edges led to a uniform heating of pulp positions 1 and 2, situated near the grinding head.

With regard to the mass of the material removed in comparison to the rotational speed chosen, it was found that doubling the rotational speed meant halving the time span of critical temperature increase in maxillary CT. This time decreased, by two thirds, when grinding mandibular CT. The mass removal experienced an increase of only 170% over all CT. In view of its effectiveness, it seems more sensible to work with a lower rotational speed. The considerable variances in the mass removal of tooth substance in individual teeth may be explained by the individual firmness of the tooth’s hard substance. In hypsodont equine teeth, the degree of mineralization of dental enamel varies between 77% and 89% [6,20], while brachyodont teeth show a homogenous degree of mineralization of 96% to 98% [21]. Human dentistry suspects a softening of enamel caused by acids to be responsible for the difference in tooth material loss under similar grinding conditions. Acidic food is mentioned as an extrinsic factor, which, especially in vegetarians, leads to an accumulated softening of hard tooth substance [22]. The use of feedstuff (grass, hay, silage) with different pH-values may possibly cause inter-individual differences in the hardness of tooth substances, too.

## Conclusion

The use of artificially normed hypsodont CT enables comparative studies of temperature increase within the pulp cavities during the grinding of occlusal tooth surfaces. A standardization of influencing parameters (e.g. force applied, rotational speeds, roughness of grinding disk, etc.) is necessary to standardize the influential factors and to issue recommendations of how to avoid iatrogenic pulp damage. In any case, the anatomical structure of the natural vital hypsodont tooth must be kept in mind, so that the findings of this study do not create a deceptive sense of security with regard to the time-dependent heating of the native pulp.

## Competing interests

None of the authors has any financial or non-financial competing interest which could inappropriately influence or bias the content of the paper.

## Authors’ contributions

SH designed the study, performed measurements, analyzed data, drafted and wrote the manuscript. ML contributed to the study design, data analysis and interpretation. HS contributed to the study design. CS contributed to the study design, data analysis and interpretation and helped with editing. All authors read and approved the final manuscript.
